# Frequency and Associated Factors of Bone Fractures in Russians: The Ural Eye and Medical Study

**DOI:** 10.1038/s41598-018-25928-1

**Published:** 2018-05-10

**Authors:** Mukharram M. Bikbov, Rinat R. Fayzrakhmanov, Gyulli M. Kazakbaeva, Rinat M. Zainullin, Venera F. Salavatova, Timur R. Gilmanshin, Inga I. Arslangareeva, Nikolai A. Nikitin, Songhomitra Panda-Jonas, Svetlana R. Mukhamadieva, Dilya F. Yakupova, Renat I. Khikmatullin, Said K. Aminev, Ildar F. Nuriev, Artur F. Zaynetdinov, Yulia V. Uzianbaeva, Jost B. Jonas

**Affiliations:** 1 Ufa Eye Research Institute, Ufa, Bashkortostan Russia; 2 Department of Ophthalmology, Medical Faculty Mannheim of the Ruprecht-Karls-University of Heidelberg, Heidelberg, Germany

## Abstract

With information about frequency of bone fractures in Russia mostly missing, we assessed the frequency of previous bone fractures in a Russian population. The population-based study Ural Eye and Medical Study included 5899 (80.5%) out of 7328 eligible individuals (mean age: 59.0 ± 10.7 years; range: 40–94 years). The history of previous bone fractures was assessed in a standardized interview for 5397 (91.5%) individuals. Mean frequency of any previous bone fracture was 1650/5397 (30.6%; 95% confidence interval (CI): 29, 3, 31.8). In multivariate analysis, higher frequency of bone fractures was associated with male sex (*P* < 0.001; odds ratio (OR): 1.67; 95% CI: 1.41, 2.00), urban region (*P* < 0.001; OR: 1.45; 95% CI: 1.23, 1.72), higher prevalence of vigorous activity during leisure (*P* < 0.001; OR: 1.42; 95% CI: 1.20, 1.68), current smoking (*P* = 0.001; OR: 1.46; 95% CI: 1.16, 1.82) and higher prevalence of cardiovascular disease (*P* = 0.007; OR: 1.29; 95% CI: 1.07, 1.56), low blood pressure episodes with hospital admission (*P* = 0.001; OR: 2.08; 95% CI: 1.37, 3.16), tumbling (*P* < 0.001; OR: 2.58; 95% CI: 1.37, 3.16) and thoracic spine pain (*P* < 0.001; OR: 1.43; 95% CI: 1.18, 1.73). In women, menopause (*P* < 0.001; OR: 2.17; 95% CI: 1.47, 3.22) was additionally associated. The most common single-bone fractures involved leg and knee (229/5397; 4.2%), hand in general (n = 169; 3.1%) or hand wrist only (n = 97; 1.8%), arm (n = 94; 1.7%) and ankle (n = 67; 1.2%). Severe fractures included spine (n = 35; 0.6%), os sacrum (n = 10; 0.2%), skull (n = 6; 0.1%), pelvis (n = 5; 0.1%) and hip (n = 22; 0.4%). Most frequent combined fractures included as most important part the leg (n = 90; 1.7%), spine (n = 18; 0.3%), and hip (n = 18; 0.3). These data give hints on the epidemiology of bone fractures in Russia.

## Introduction

Bone fractures can lead to severe functional deficits and are an important element in the causes of disability and burden of disease in all world regions^1^. Although not specifically analyzed as bone fractures, unintentional injuries and transport injuries each showed from 1990 to 2015 a decrease in their age-standardised DALY (disability-adjusted life years) rates by 20% and 17%, respectively, in the Global Burden of Disease Study (GBD) 2015^2^. Despite the broad decreases in age-standardised rates of injury burden, however, the pace of progress for these causes of DALYs has been comparatively slow and ultimately has led to minimal changes in the proportion of overall burden due to injuries during the past 25 years^1,2^. The total injury DALYs remained mostly unchanged between 1990 and 2015 and counted in the year 2015 for 249, 791 DALYs (95% uncertainty interval (UI): 231, 409 to 266, 419) or 10.1% out of a total of 2,464,895 (95% UI: 2,259,889 to 2,696,510)^2^.

Despite their importance for public health, studies which have assessed the frequency of bone fractures in a population-based manner, globally or for Russia, have been scarce^3–14^. Since bone fractures are at least partially preventable and since they are the cause for a major fraction of DALYs worldwide, we conducted this study to assess the frequency of previous bone fractures in a population in Russia and explored associations of previous bone fractures with other parameters such as sex, region of habitation and level of education.

## Methods

The Ural Eye and Medical Study (UEMS) is a population-based study which was carried out in the urban region of Kirovskii of the city of Ufa and in villages of the rural region of the Karmaskalinsky District in a distance of 65 km from Ufa^15^. According to the Declaration of Helsinki, the Ethics Committee of the Academic Council of the Ufa Eye Research Institute approved the study and all participants gave informed written consent. The ethics committee confirmed that all methods were performed in accordance with the relevant guidelines and regulations. Ufa as the capital of the republic of Bashkortostan in Russia is an industrial, economic, scientific and cultural center. The Republic of Bashkortostan with a population of about 4.07 million people is located in the west of the southern Ural Mountains about 1300 km East of Moscow. With altogether 1.1 million inhabitants, the citizenship of Ufa is ethnically composed of Russians, Tatars, Bashkirs, Ukrainians and other ethnicities^16^. Inclusion criterion for the participation in the study was living in the study region and having an age of 40+ years. There were no exclusion criteria.

Trained social workers performed a standardized interview including more than 250 questions on socioeconomic parameters such as level of education, family income and family possessions, living conditions (such as toilet availability in the house, lighting source, agricultural land and livestock ownership, size of the family), diet (such as frequency and amount of intake of vegetables, fruits and meat), smoking or other types of tobacco consumption, daily physical activity, alcohol consumption, depression and suicidal ideas, and medical history including known diagnosis and therapy of major diseases such as arterial hypertension, diabetes mellitus, and cardiovascular diseases. The interview included standardized questions which had been validated in previous studies such as the mini–mental state examination or Folstein test or in Zung’s self-rated depression scale^17,18^. The questionnaire included in particular questions on any previous bone fracture and which bones were involved. We collected and reported the data using the Guidelines for Accurate and Transparent Health Estimates Reporting (GATHER statement guidelines)^19^.

For all study participants, we measured the arterial blood pressure and pulse rate and the anthropomorphic parameters of body height, body weight and circumference of the hip and waist. We calculated the body mass index (BMI) as ratio of body weight (measured in kilogram) divided by the square of the body height (measured in meters). The handgrip strength was determined using a dynamometer (dynamometer - dk 140, ZAO Nizhnetagilskiy Medical Instrument Plant, Nizhniy Tagil, Russia). Hearing loss was examined performing Rinne’s test and Weber’s test. The examination of blood samples, taken under fasting conditions consisted of a complete blood count and measurement of the serum concentrations of glucose, blood lipids, C-reactive protein, erythrocyte sedimentation rate, hemoglobin, blood clotting time, total protein, bilirubin, urea, nitrogen, creatinine, prothrombin index, alanine transaminase, aspartate transaminase, and rheumatoid factor (devices used were a photoelectrocolorimeter (KFK-3, ≪Zagorsk optical and mechanical plant≫, Zagorsk, Russia), Abbe refractometer (IRF-45, Kazan optical and mechanical plant, Kazan, Russia), coagulometer (ASK 2-01 Astra, NPZ Astra, Ufa, Russia) and semiautobiochemical analyzer (BS-3000P, Sinnowa, Nanjing, China)). All participants underwent a pulmonary function test by spirometry (Riester spirotest, Riester Company, Jungingen, Germany). Chronic obstructive pulmonary disease (COPD) was defined by a cut-off value of the ratio of forced expiratory volume in one second (FEV1) divided by the forced vital capacity (FVC) of less than 0.7. Asthma was defined by a self-reported diagnosis of physician-made diagnosis of asthma. We defined arterial hypertension by a systolic blood pressure ≥140 mmHg and/or a diastolic blood pressure ≥90 mmHg, and/or self-reported history or current treatment of arterial hypertension with antihypertensive medication. A glucose concentration ≥7.0 mmol/L or a self-reported history of physician diagnosis of diabetes mellitus or a history of drug treatment for diabetes (insulin or oral hypoglycemic agents) characterized the presence of diabetes mellitus. Depression was assessed applying the Center for Epidemiologic Studies Depression Scale (CES-D) Scoresheet. The study design has been described in detail recently^15^.

We used a commercially available statistical software program (Statistical Package for Social Science, SPSS, version 22.0; IBM-SPSS Inc., Chicago, USA) for statistical analysis. In a first step, we determined the frequency of a positive history of previous bone fractures, presenting the results as mean and 95% confidence intervals (CI). In a second step, we searched for associations in univariate analysis between the frequency a positive history of previous bone fractures and other parameters. In a third step, we conducted a multivariate binary regression analysis with the frequency of a positive history of previous bone fractures as dependent variable and as independent variables all those parameters which were significantly associated with previous bone fractures in the univariate analysis. All variables in the list of independent parameters were tested for muliticollinearity. Odds ratios (OR) and their 95% confidence intervals (CI) were calculated. All P-values were two-sided and considered statistically significant when the values were less than 0.05.

### Data availability

The data as basis for this study are available at: 10.6084/m9.figshare.6168839.

## Results

The study included 5899 individuals (2580 (43.7%) men) out of a population of 7328 eligible individuals, who resided in the study regions and who fulfilled the inclusion criterion of an age of 40+ years. The participation rate as ratio of 5899/7328 was 80.5%. Out of the 5889 individuals primarily participating in the Ural Eye and Medical Study, the present study included 5397 (91.5%) individuals with available information on the history of previous bone fractures. The mean age of the study population (2450 (45.4%) men) was 58.6 ± 10.6 years (median: 58 years; range: 40–94 years). The composition of the study population with respect to sex and age corresponded to the sex and age distribution in the Russian population according to the most recent census carried out in 2010^20^. The mean body height was 165.0 ± 8.8 cm (median: 164 cm; range: 112–196 cm), the mean body weight was 75.9 ± 14.6 kg (median: 75 kg; range: 31–170 kg), and the mean body mass index was 27.9 ± 5.0 kg/m^2^ (median: 27.3 kg/m^2^; range: 13.96–60.96 kg/m^2^). The mean waist circumference was 93.8 ± 13.3 cm (median: 94 cm; range: 45–158 cm), and the mean hip circumference was 103.2 ± 12.6 cm (median: 103 cm; range: 45–194 cm). Illiteracy was present for 14 (0.3%) individuals, 86 (1.6%) participants had passed the fifth grade, 557 (10.3%) participants the 8th grade, 628 (11.6%) participants the 10th grade, and 638 (11.8) individuals the 11th grade. Graduates were 1785 (33.1%) individuals, and post graduates were 51 (0.9%) study participants, and 1634 individuals had a specialized secondary education. Among the study population, 6 (0.1%) subjects took a vegetarian diet, while the remaining individuals had a mixed diet. Mean systolic blood pressure was 133.0 ± 20.0 mmHg (range: 83–225 mmHg), and mean diastolic blood pressure was 82.1 ± 10.3 mm Hg (range: 40–154 mm Hg). Known and treated arterial hypertension was present in 2129 (39.5%) of the study participants.

The group of individuals with information on previous bone fractures as compared with the group of subjects without bone fracture data was significantly younger (58.6 ± 10.6 years versus 63.0 ± 11.3 years; *P* < 0.001), and had significantly more men (2450 (45.4%) men/2947 (54.6%) women) versus (130 (25.9%) men/372 (74.1%) women); *P* < 0.001.

Mean frequency of any previous bone fracture was 1650/5397 (30.6%; 95% CI: 29.3, 31.8) (Table 1). Within the ethnically Russian group (n = 1185; 508 men, 677 women) with a mean age of 60.1 ± 11.1 years, the frequency of bone fractures was 408/1185 (34.4%; 95% CI: 31.7, 37.1). In univariate analysis, the frequency of bone fractures was significantly higher in the Russian group than in the non-Russian group (*P* = 0.001; OR: 1.26; 95% CI: 1.10, 1.44).

**Table 1:** Frequency (%) of previous bone fractures in the Ural Eye and Medical Study, stratified by sex and age.

Age Group (Years)	n	Previous Bone Fractures (%)	95% Confidence Intervals
Men			
40–44	209	42.1	35.4, 48.9
45–49	350	38.0	32.9, 43.1
50–54	422	38.2	33.5, 42.8
55–59	471	34.0	29.7, 38.3
60–64	383	36.6	31.7, 41.4
65–69	273	35.9	30.2, 41.6
70–74	124	31.5	23.2, 39.7
75–79	150	30.7	23.2, 38.1
80+	68	27.9	17.0, 38.9
Women			
40–44	252	17.5	12.7, 22.2
45–49	361	16.6	12.8, 20.5
50–54	465	18.7	15.2, 22.3
55–59	500	22.6	18.9, 26.3
60–64	449	30.7	26.5, 35.0
65–69	430	32.6	28.1, 37.0
70–74	182	45.1	37.8, 52.4
75–79	203	32.5	26.0, 39.0
80+	105	34.3	25.1, 43.5

Among the bone fractures, the most frequent fractures with involvement of a single bone were fractures of the lower extremities including the upper or lower leg and the knee (n = 229; 229/5397 or 4.2%), followed by fractures of the upper extremity including the arm (n = 94; 1.7%) (Table 2). The most frequent combined fractures with involvement of at least two bones were fractures with the leg as the most important part (n = 90; 1.7%) (Table 2).

**Table 2:** Frequency (%) of previous bone fractures in the Ural Eye and Medical Study, stratified by fracture location.

Previous Bone Fracture Type	n	Percentage of the Study Population (%)
Single bone fractures, lower extremity		
Knee	229	4.2
Ankle	67	1.2
Foot and Heel	54	1.0
Toes	22	0.4
Single bone fractures, upper extremity		
Arm	94	1.7
Elbow	13	0.2
Hand Wrist Only	97	1.8
Fingers Only	64	1.2
Hand	169	3.1
Clavicle	89	1.6
Shoulder	18	0.3
Single bone fractures, other bones		
Rips	85	1.6
Hip	22	0.4
Nose	12	0.2
Chin	18	0.3
Spine	35	0.6
Skull	6	0.1
Pelvis	5	0.1
Os sacrum	10	0.2
Combined fractures, with the most important fracture of:		
Leg	90	1.7
Hand	23	0.4
Arm	9	0.2
Wrist	9	0.2
Shoulder	8	0.1
Ankle	8	0.1
Pelvis	3	0.1
Others	316	5.9

**Figure 1:**
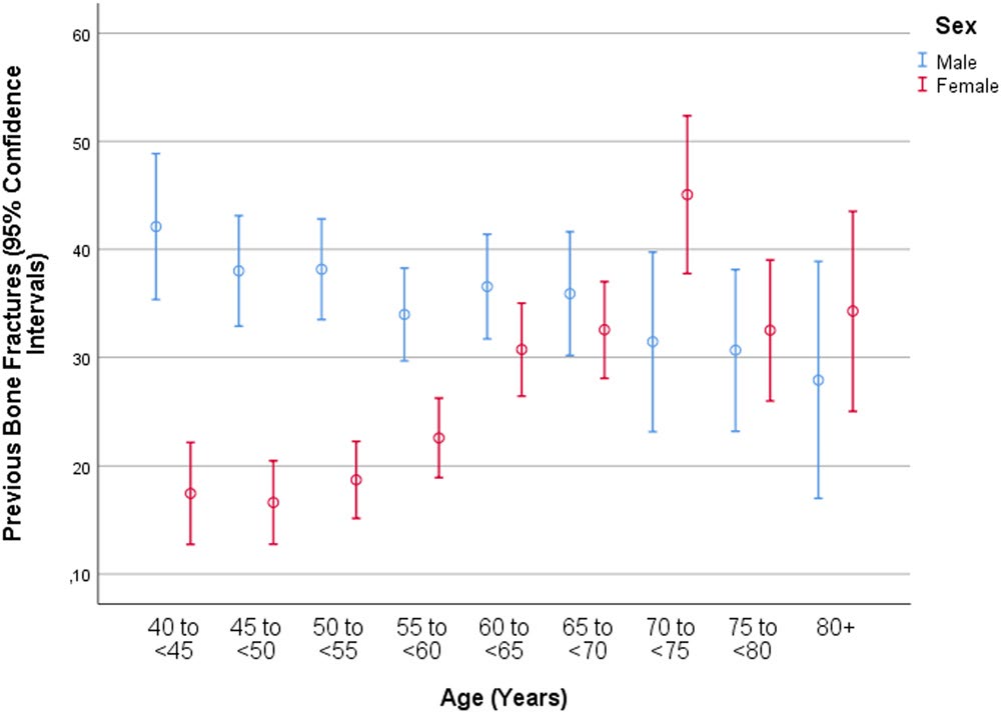
Graph showing the frequency of previous bone fractures stratified by age and sex in the Ural Eye and Medical Study.

In univariate analysis, the frequency of bone fractures was correlated with parameters such as older age (*P* < 0.001) (Fig. 1), male sex (*P* < 0.001) (Fig. 1), urban region of habitation (*P* < 0.001) and others (Table 3). The binary regression analysis included the positive history of previous bone fractures as dependent variable and as independent variables all those parameters which were significantly associated with the occurrence of bone fractures in the univariate analysis. Due to collinearity, we first dropped body hip circumference and prevalence of daily smoking. Due to lack of statistical significance, we then dropped step by step Russian ethnicity (*P* = 0.99), age (*P* = 0.98), waist-hip circumference ratio (*P* = 0.89), hearing loss score (*P* = 0.97), ownership of a two-wheeler (*P* = 0.86), hand grip dynamometric force (*P* = 0.75), smoking package year (*P* = 0.66), history of osteoarthritis (*P* = 0.53) and arthiritis (*P* = 0.55), body height (*P* = 0.35), depression score (*P* = 0.36), blood hemoglobin concentration (*P* = 0.31), number of days with intake of vegetables (*P* = 0.30) and fruits (*P* = 0.46), history of low back pain (*P* = 0.30), history of unconsciousness (*P* = 0.29) and demention (*P* = 0.20), consumption of alcohol (*P* = 0.17), body mass index (*P* = 0.12), prevalence of chronic obstructive pulmonary disease (*P* = 0.11), diastolic blood pressure (*P* = 0.13), history of any ocular disease (*P* = 0.16), and type of meat processing (*P* = 0.05). In the resulting final model, a higher prevalence of bone fractures was associated with male sex (*P* < 0.001), urban region of habitation (*P* < 0.001), higher prevalence of vigorous activity during leisure time (*P* < 0.001), current smoking (*P* = 0.001), lower prevalence of ownership of a laptop (*P* = 0.006), and higher prevalence of a positive history of cardiovascular disease (*P* = 0.007), low blood pressure episodes with hospital admission (*P* = 0.001), tumbling (*P* < 0.001) and thoracic spine pain (*P* < 0.001) (Table 4). If the parameter of a history of menopause was added to the model in women, it was significantly associated (*P* < 0.001; OR: 2.17; 95% CI: 1.47, 3.22), while history of thoracic spine pain (*P* = 0.94), cardiovascular disease (*P* = 0.11) and low blood pressure episodes with hospital admission (*P* = 0.07) were no longer significantly associated with the frequency of previous bone fractures.

**Table 3:** Associations between the frequency (%) of previous bone fractures in the Ural Eye and Medical Study and other systemic parameters.

Parameter	*P*-Value	Odds Ratio	95% Confidence Interval
Age (Years)	<0.001	1.01	1.02, 1.02
Sex: men/women	<0.001	0.62	0.55, 0.70
Urban/rural region of habitation	<0.001	0.67	0.60, 0.75
Family status: Married versus any other status	0.66	—	—
Family type: Joint (three generations)/nuclear (two generations)/single/family of 2 people	0.37	—	—
Ethnicity: Russian/any other ethnicity	0.001	1.26	1.10, 1.44
Body height (cm)	<0.001	1.02	1.01, 1.03
Body weight (kg)	0.81	—	—
Body mass index (kg/m^2^)	0.004	0.98	0.97, 0.99
Waist circumference (cm)	0.49	—	—
Hip circumference cm)	0.02	0.995	0.990, 0.999
Waist-Hip-Ratio	<0.001	2.90	1.55, 5.42
Socioeconomic parameters			
Level of education	0.10	0.97	0.94, 1.01
Monthly Income (Below poverty line/average/above average/high)	0.40	—	—
Own ownership of house (yes/no)	0.42	—	—
Own ownership of refrigerator (yes/no)	0.57	—	—
Own ownership of second house (yes/no)	0.45	—	—
Own ownership of telephone (yes/no)	0.08	1.17	0.98, 1.40
Own ownership of smartphone (yes/no)	0.77	—	—
Own ownership of television set (yes/no)	0.40	—	—
Own ownership of car (yes/no)	0.08	0.87	0.74, 1.02
Own ownership of two-wheeler (yes/no)	0.01	0.86	0.77, 0.97
Own ownership of tractor (yes/no)	0.47	—	—
Own ownership of bullock cart (yes/no)	0.45	—	—
Own ownership of computer/laptop (yes/no)	0.03	0.83	0.71, 0.98
Physical activity			
How long is your usual work day? (Minutes)	0.90	—	—
Does your work involve mostly sitting or standing with less than 10 minutes of walking at a time? (Yes/No)	0.09	0.89	0.78, 1.02
Does your work involve physically vigorous activity (like heavy lifting or digging) or physically moderate intensity activity (like brisk walking or carrying light loads) (Yes/No)	0.36	—	—
How many days a week do you do such physically vigorous activity during work?	0.48	—	—
On a usual day how much time do you spend on such physically vigorous work during work? (Minutes)	0.94	—	—
Does your work involve physically moderate-intensive activity, like brisk walking or carrying light loads for at least 10 minutes at a time?	0.44	—	—
In a typical week, on how many days do you walk or use a bicycle (pedal cycle) for at least 10 minutes continuously to get to and from places?	0.34	—	—
In your leisure time, do you do any physically vigorous activities like running, strenuous sports or weight lifting for at least 10 minutes at a time?	<0.001	1.31	1.15, 1.49
In a typical week, on how many days do you do physically moderately intensive activities like brisk walking, cycling or swimming for at least 10 minutes at a time in your leisure time?	0.87	—	—
Over the past 7 days, how much time did you spend sitting or reclining on a typical day?	0.85	—	—
History of diseases			
History of angina pectoris	0.74	—	—
History of asthma	0.13	—	—
History of arterial hypertension	0.11	—	—
History of arthritis	<0.001	1.31	1.15, 1.49
History of low back pain	<0.001	1.28	1.13, 1.43
History of thoracic spine pain	<0.001	1.37	1.20, 1.56
History of neck pain	0.90	—	—
History of headache	0.34	—	—
History of cancer	0.93	—	—
History of cardiovascular disorders including stroke	<0.001	1.30	1.15, 1.48
History of dementia	0.02	2.16	1.13, 4.13
History of diabetes mellitus	0.09	1.20	0.98, 1.47
History of diarrhea	0.47	—	—
History of iron-deficiency anemia	0.89	—	—
History of low blood pressure and hospital admittance	<0.001	1.88	2.39, 2.54
History of osteoarthritis	0.009	1.22	1.05, 1.41
History of skin disease	0.22	—	—
History of thyreopathy	0.36	—	—
History of tumbling	<0.001	2.67	2.33, 3.07
History of unconsciousness	0.03	1.26	1.03, 1.54
Age of the last menstrual bleeding (years)	0.20	—	—
Age of last regular menstrual bleeding (years)	0.21	—	—
History of menopause	<0.001	2.24	1.75, 2.87
Blood concentrations (mmol/L) of:			
Alanine aminotransferase (IU/L)	0.12	—	—
Aspartate aminotransferase (IU/L)	0.66	—	—
Bilirubin, total (µmol/L)	0.29	—	—
High-density lipoproteins (mmol/L)	0.91	—	—
Low-density lipoproteins (mmol/L)	0.49	—	—
Cholesterol (mmol/L)	0.93	—	—
C-reactive protein (mg/L)	0.06	0.83	0.68, 1.01
Rheumatoid factor (IU/mL)	0.32	—	—
Erythrocyte sedimentation rate (mm/hour)	0.22	—	—
Glucose (mmol/L)	0.38	—	—
Prevalence of diabetes mellitus	0.07	1.19	0.99, 1.43
Creatinine (µmol/L)	0.70	—	—
Urea (mmol/L)	0.19	—	—
Residual nitrogen (g/L)	0.15	—	—
Total protein (g/L)	0.99	—	—
International normalized ratio (INR)	0.91	—	—
Prothrombin time (%)	0.60	—	—
Hemoglobin	0.01	1.01	1.001, 1.01
Erythrocytes (10^6^ cells/µL)	0.11	—	—
Leukocytes (10^9^ cells/L)	0.50	—	—
Rod-core granulocyte (% of leukocytes)	0.98	—	—
Segment nuclear granulocyte (% of leukocytes)	0.21	—	—
Eosinophil granulocytes (% of leukocytes)	0.74	—	—
Lymphocytes (% of leukocytes)	0.29	—	—
Monocytes (% of leukocytes)	0.21	—	—
Blood pressure, systolic (mmHg)	0.06	1.003	1.000, 1.006
Blood pressure, diastolic (mmHg)	0.003	1.01	1.003, 1.01
Blood pressure, mean (mmHg)	0.11	—	—
Prevalence of arterial hypertension	0.17	—	—
Prevalence of chronic obstructive pulmonary disease	<0.001	1.63	1.31, 2.03
Diet			
Vegetarian diet/mixed diet	0.88	—	—
Number of meals per day	0.12	—	—
In a week how many days do you eat fruits?	0.004	0.96	0.93, 0.99
In a week how many days do you eat vegetables?	0.03	0.95	0.92, 0.99
Type of oil used for cooking: vegetable oil/non-vegetable oil	0.62	—	—
Salt consumed per day (g)	0.45	—	—
Degree of processing of meat (weak/medium/well done)	0.02	1.14	1.02, 1.27
Smoking			
Do you currently smoke any tobacco products? (yes)	<0.001	1.86	1.58, 2.19
Do you smoke daily? (yes/no)	<0.001	1.90	1.62, 2.25
Package years (package = 20 cigarettes)	<0.001	1.02	1.01, 1.02
Alcohol			
Alcohol consumed such as beer, whisky, rum, gin brandy or other local products? (yes/no)	0.01	1.20	1.05, 1.37
How many alcoholic drinks do you have on a typical day when you are drinking)	0.13	—	—
How often do you have 6 or more drinks on one occasion? (never/rarely/sometimes/often/cannot say)	0.12	—	—
Hearing loss			
Hearing Loss Total Score	0.02	1.01	1.001, 1.01
Depression			
Depression score	0.003	1.02	1.01, 1.04
State-Trait Anxiety Inventory (STAI)			
Anxiety score	0.17	—	—
Dynamometry			
Manual dynamometry, right hand (dekaNewton)	0.001	1.01	1.003, 1.01
Manual dynamometry, left hand (dekaNewton)	0.001	1.02	1.01

**Table 4:** Associations (multivariate analysis) of the frequency of previous bone fractures in the Ural Eye and Medical Study.

Parameter	*P*-Value	Odds Ratio	95% Confidence Interval
Sex (Men/Women)	<0.001	1.67	1.41, 2.00
Region of Habitation (Urban/Rural)	<0.001	1.45	1.23, 1.72
Vigorous Activity during Leisure Time	<0.001	1.42	1.20, 1.68
Current Smoking	0.001	1.46	1.16, 1.82
Ownership of a Laptop	0.006	0.79	0.67, 1.82
History of Cardiovascular Disease	0.007	1.29	1.07, 1.56
History of Low Blood Pressure Episode with Hospital Admission	0.001	2.08	1.37, 3.16
History of Tumbling	<0.001	2.58	2.11, 3.15
History of Thoracic Spine Pain	<0.001	1.43	1.18, 1.73

If only individuals with an age of more than 60 years were taken into account, in attempt to focus on osteoporosis-related bone fractures, the parameters of history of intake of steroids (*P* = 0.62), alcohol consumption (*P* = 0.33), body weight (*P* = 0.34) and body height (*P* = 0.94) were not significantly associated with the frequency of previous bone fractures. Similar findings were obtrained for cut-off values of an age of 65 years and of 70 years. The group of individuals with hip fractures was too small for a separate meaningful multivariate analysis.

## Discussion

In this population-based study, about one third of the population with a mean age of 58.6 ± 10.6 years had previously suffered a bone fracture. A higher frequency of a previous bone fractures was associated with male gender, urban region of habitation, higher prevalence of vigorous activity during leisure time, current smoking, lower prevalence of ownership of a laptop and higher prevalence of a positive history of cardiovascular disease, low blood pressure episodes with hospital admission, tumbling and thoracic spine pain. In women, history of menopause was additionally associated with the history of bone fractures. With only a single bone involved, the most common fractures were fractures of the lower extremities followed by fractures of the upper extremity. Severe fractures included the spine (35/5397 or 0.6%), skull (n = 6/5397 or 0.1%), pelvis (n = 5/5397 or 0.1%), and os sacrum (n = 10/5397 or 1.9%). The most frequent combined fractures with involvement of at least two bones were fractures with the leg as the most important part (n = 90/5397 or 1.7%). Hip fractures, as single bone fracture (n = 22; 0.4%) or in combination with the fracture of other bones (n = 18; 0.3%) had occurred in 40 (0.7%) of the study participants.

These data agree with the results of previous investigations^5–7^. In a retrospective study by Mikhaĭlov and colleagues in two cities in Russia, the incidence of hip fractures was 61 per 100,000 person-years (32 for men, and 77 for women) and 61 per/100,000 person-years (45 for men and 70 for women), resp.^5^. The risk of hip fractures increased with older age. As in our study, the study by Mikhaĭlov and colleagues showed that the incidence of hip fractures was lower in Russia than in other European countries. In the study by Lesnyak and associates, the incidence of index fractures increased with older age and showed a female preponderance^6^. The lifetime probability of a hip fracture at the age of 50 years was 4% in men and 7% in women. While the risk factors for bone fractures, i.e. older age and female sex, agreed with those found in our study, the incidence figures in Lesnyak´s study and the results obtained in our study cannot directly be compared. The findings obtained in our study also agree with the results of the fracture risk assessment studies (FRAX) in that smoking was a risk factor for bone fractures^8–16^. In partial agreement with the FRAX studies, alcohol consumption and history of osteoarthritis were associated with a higher frequency of previous bone farctures in univariate analysis.

The figures on fractures obtained in our study population were lower than those reported in studies on Western populations. Using the data collected by the Swiss Federal Office of Statistics for hospitalised fractured patients, Lippuner and colleagues reported that major osteoporotic fractures showed an incidence of 773 per 100,000 men and and 2,078 per 100,000 women^6^. For a person at the age of 50 years, the lifetime probability for a future major osteoporotic fracture was estimated to be 20.2% for men and 51.3% for women. As in our study the risk of osteoporotic fractures increased with older age and it was higher in women than in men. In a similar study performed in Colombia by Jaller-Raad and coworkers, individuals at the age of 50 years had a cumulative probability of a future hip fracture of 2.5 in men and 4.7% in women. These figures were lower than those for a Mexican population (3.8 and 8.5%, resp.) and they were comparable with the estimates for a study population from Venezuela (2.4 and 7.5%, resp.)^7^. Similar results were obtained for the Dutch population by Lalmohamed and associates^9^. In the Japanese investigation by Sakuma and colleagues, the incidence of fractures of the vertebra was 233 per a population of 100,000, and it was 121 per 100,000 for fractures of the hip, 109 for fractures of the distal radius, and 37 for fractures of the proximal humerus^12^. Combined, these four fracture types had an incidence of was 500 per 100,000 persons per year. The average age when the fractures occurred was 81 years for fractures of the hip, 78 years for fractures of the vertebra, 76 for fractures of the proximal humerus, and 60 years for fractures of the distal radius.

The results obtained in the present study indicate that the frequency of bone fractures in our study population was lower than the frequency of bone fractures as reported in studies on Western populations. The reasons for the discrepancy may be due to differences in the lifestyle, the diet, the amount and type of physical activity, and other factors. To cite an example, the frequency of previous bone fractures was lower in the rural population than in the urban population in our study, with the rural population being mostly farmers having a higher amount of continuous physical activity in their daily life. Physical exercise has been shown to reduce fall-related bone fractures^21^. Future studies may further explore other factors associated with the discrepancy in the frequency of bone fractures between the population of our study and Western populations.

Limitations of our study should be mentioned. First, the assessment of a previous bone fracture depended on the answers made in the standardized interviews. Minor injuries with bone fractures which did not need medical therapy (i.e. toe fractures) or which did not undergo medical treatment might not have been mentioned. Also, the cognitive functions of some of the older participants of the study population might have been reduced, so that these participants might not have fully remembered all bone fractures. Age-related loss of memory however mostly affects the short-term memory so that bone fractures, which occured several years before the interview was conducted, might have well been remembered. Second, as for any population-based study the participation rate and the representativeness of the study population as compared to the population of the region or country the study is aiming at is critical. In our study, 80.5% of the eligible population participated in the survey so that a major bias in the inclusion of study participants may appear unlikely. The region of the study, a major city and a rural region in the Southern Russian republic of Bashkortostan West of the Ural Mountains was typical for the whole region of Southern Russia. Despite its relatively southern location, its continental climate with cold, harsh and long winters and warm to hot summers is relatively comparable with the continental climate in North-Western Russia and Central Russia. The multi-ethnic composition of our study population was typical for Southern Russia and showed as compared to North-Western Russia and Central Russia a lower percentage of Russians on the total population. To overcome this limitation, we assessed the frequency of previous bone fractures in dependence of the ethnic background and found, that the prevalences did not differ significantly between the Russian groups than in the non-Russian group. The age and sex distribution in our study population was comparable to the results of the Russian census 2010.

In conclusion, in this typically ethically mixed urban and rural Russian population aged 40+ years, the mean frequency of any previous bone fracture was 30.6% (95% CI: 29, 3, 31.8), with the associated factors of male sex, urban region of habitation, higher prevalence of vigorous activity during leisure time, current smoking, lower prevalence of ownership of a laptop and higher prevalence of a positive history of cardiovascular disease, low blood pressure episodes with hospital admission, tumbling and thoracic spine pain, as well as history of menopause in women. The most common single-bone fractures involved leg and knee, hand and arm. These data show the epidemiology of bone fractures in Russia.

## References

[1] GBD 2016 DALYs and HALE Collaborators (2017). Global, regional, and national disability-adjusted life-years (DALYs) for 333 diseases and injuries and healthy life expectancy (HALE) for 195 countries and territories, 1990–2016: a systematic analysis for the Global Burden of Disease Study 2016. Lancet..

[2] Kassebaum NJ (2016). Global, regional, and national disability‐adjusted life years (DALYs) for 315 diseases and injuries and healthy life expectancy (HALE) for 195 countries and territories, 1990–2015: a systematic analysis for the Global Burden of Diseases, Injuries, and Risk Factors (GBD) 2015 Study. Lancet..

[3] Mikhaĭlov EE, Benevolenskaia LI, Ershova OB, Bobylev VI (1995). [Epidemiology of hip fractures in age groups at high risk for osteoporosis]. Ter Arkh..

[4] Lesnyak O (2012). Epidemiology of fracture in the Russian Federation and the development of a FRAX model. Arch Osteoporos..

[5] Ershova OB, Bobylev VIA, Semenova OV, Belosel’skiĭ NN (2012). [The epidemiology of osteoporotic fractures in the population of the city of Yaroslavl]. Ter Arkh. X.

[6] Lippuner K, Johansson H, Kanis JA, Rizzoli R (2009). Remaining lifetime and absolute 10-year probabilities of osteoporotic fracture in Swiss men and women. Osteoporos Int..

[7] Jaller-Raad JJ (2013). Incidence of hip fracture in Barranquilla, Colombia, and the development of a Colombian FRAX model. Calcif Tissue Int..

[8] Maravic M, Le Bihan C, Landais P, Fardellone P (2005). Incidence and cost of osteoporotic fractures in France during 2001. A methodological approach by the national hospital database. Osteoporos Int..

[9] Lalmohamed A (2012). Calibration of FRAX ® 3.1 to the Dutch population with data on the epidemiology of hip fractures. Osteoporos Int..

[10] Zerbini CA (2015). Incidence of hip fracture in Brazil and the development of a FRAX model. Arch Osteoporos..

[11] Stepan JJ (2012). Hip fracture incidence from 1981 to 2009 in the Czech Republic as a basis of the country-specific FRAX model. Calcif Tissue Int..

[12] Sakuma M (2008). Incidence and outcome of osteoporotic fractures in 2004 in Sado City, Niigata Prefecture, Japan. J Bone Miner Metab..

[13] Nguyen TV, Center JR, Sambrook PN, Eisman JA (2001). Risk factors for proximal humerus, forearm, and wrist fractures in elderly men and women: the Dubbo Osteoporosis Epidemiology Study. Am J Epidemiol..

[14] Wade SW, Strader C, Fitzpatrick LA, Anthony MS (2012). Sex- and age-specific incidence of non-traumatic fractures in selected industrialized countries. Arch Osteoporos..

[15] Bikbov, M. M., Fayzrakhmanov, R. R., Kazakbaeva, G. M. & Jonas, J. B. The Ural Eye and Medical Study: Description of study design and methodology. *Ophthalmic Epidemiol*. 1–12. 10.1080/09286586.2017.1384504. [Epub ahead of print] 2017 Oct 9. 10.1080/09286586.2017.1384504 28990827

[16] Wikipedia. Bashkortostan. https://en.wikipedia.org/wiki/Bashkortostan; assessed 4.3.2018.

[17] Folstein MF, Folstein SE, McHugh PR (1975). “Mini-mental state”. A practical method for grading the cognitive state of patients for the clinician. J Psychiatr Res..

[18] Zung WW (1965). A Self-rating depression scale. Arch Gen Psychiatry..

[19] Stevens GA (2016). Guidelines for Accurate and Transparent Health Estimates Reporting: the GATHER statement. Lancet..

[20] https://en.wikipedia.org/wiki/Demographics_of_Russia. Assessed 5.11.2017.

[21] de Kam D, Smulders E, Weerdesteyn V, Smits-Engelsman BC (2009). Exercise interventions to reduce fall-related fractures and their risk factors in individuals with low bone density: a systematic review of randomized controlled trials. Osteoporos Int..

